# Synergistic Effect of Bolus Exposure to Zinc Oxide Nanoparticles on Bleomycin-Induced Secretion of Pro-Fibrotic Cytokines without Lasting Fibrotic Changes in Murine Lungs

**DOI:** 10.3390/ijms16010660

**Published:** 2014-12-30

**Authors:** Wenting Wu, Gaku Ichihara, Naozumi Hashimoto, Yoshinori Hasegawa, Yasuhiko Hayashi, Saeko Tada-Oikawa, Yuka Suzuki, Jie Chang, Masashi Kato, Corina N. D’Alessandro-Gabazza, Esteban C. Gabazza, Sahoko Ichihara

**Affiliations:** 1 Department of Occupational and Environmental Health, Nagoya University Graduate School of Medicine, Nagoya 466-8550, Japan; E-Mails: wendyvvri@med.nagoya-u.ac.jp (W.W.); jie-chang@med.nagoya-u.ac.jp (J.C.); katomasa@med.nagoya-u.ac.jp (M.K.); 2 Department of Occupational and Environmental Health, Tokyo University of Science, Noda 278-8510, Japan; 3 Department of Respiratory Medicine, Nagoya University Graduate School of Medicine, Nagoya 466-8550, Japan; E-Mails: hashinao@med.nagoya-u.ac.jp (N.H.); yhasega@med.nagoya-u.ac.jp (Y.H.); 4 Department of Electrical and Electronic Engineering, Okayama University, Okayama 700-8530, Japan; E-Mail: hayashi.yasuhiko@ec.okayama-u.ac.jp; 5 Graduate School of Regional Innovation Studies, Mie University, Tsu 514-8507, Japan; E-Mails: t-saeko@innov.mie-u.ac.jp (S.T.-O.); suzujohn@yahoo.co.jp (Y.S.); saho@gene.mie-u.ac.jp (S.I.); 6 Department of Immunology, Mie University School of Medicine, Tsu 514-8507, Japan; E-Mails: dalessac@clin.medic.mie-u.ac.jp (C.N.D.-G.); gabazza@doc.medic.mie-u.ac.jp (E.C.G.)

**Keywords:** zinc oxide nanoparticles, metal fume fever, pulmonary fibrosis, bleomycin, animal model

## Abstract

Zinc oxide (ZnO) nanoparticles are widely used in various products, and the safety evaluation of this manufactured material is important. The present study investigated the inflammatory and fibrotic effects of pulmonary exposure to ZnO nanoparticles in a mouse model of pulmonary fibrosis. Pulmonary fibrosis was induced by constant subcutaneous infusion of bleomycin (BLM). Female C57BL/6Jcl mice were divided into BLM-treated and non-treated groups. In each treatment group, 0, 10, 20 or 30 µg of ZnO nanoparticles were delivered into the lungs through pharyngeal aspiration. Bronchoalveolar lavage fluid (BALF) and the lungs were sampled at Day 10 or 14 after administration. Pulmonary exposure by a single bolus of ZnO nanoparticles resulted in severe, but transient inflammatory infiltration and thickening of the alveolar septa in the lungs, along with the increase of total and differential cell counts in BLAF. The BALF level of interleukin (IL)-1β and transforming growth factor (TGF)-β was increased at Day 10 and 14, respectively. At Day 10, the synergistic effect of BLM and ZnO exposure was detected on IL-1β and monocyte chemotactic protein (MCP)-1 in BALF. The present study demonstrated the synergistic effect of pulmonary exposure to ZnO nanoparticles and subcutaneous infusion of BLM on the secretion of pro-fibrotic cytokines in the lungs.

## 1. Introduction

Concern about the safety of manufactured nanomaterials is increasing with the remarkable development in nanotechnology. This concern is based on the novel physicochemical properties and unpredictable health effects of nanoparticles, in association with the probability of occupational and environmental exposure throughout the product chain during manufacture, application and waste management [1,2]. Zinc oxide (ZnO) nanoparticles are one of the most widely-used manufactured nanomaterials in a variety of products, including cosmetics, pigments, food additives, rubber manufacturing and electronic materials, based on their UV light absorption property, photocatalysis, semi-conduction and antibacterial action [3,4,5].

The respiratory system is considered as one of the main portals of entry for nanomaterials [6,7]. Although several *in vitro* studies showed the high toxicity of ZnO nanoparticles in alveolar macrophages and lung epithelial cells [8,9,10,11,12], only a few animal studies reported the toxicity of pulmonary exposure to ZnO nanoparticles. In rats, a large number of neutrophils and high levels of lactate dehydrogenase and microprotein were found in bronchoalveolar lavage fluid (BALF) after inhalation or intratracheal instillation of ZnO nanoparticles [13]. Oxidative stress was identified based on the high levels of lipid peroxide, heme oxygenase-1 and α-tocopherol in BALF of rats exposed to ZnO nanoparticles by intratracheal instillation [14]. In addition to the above proinflammatory effects, bronchocentric interstitial fibrosis was observed at four weeks after a single instillation of ZnO nanoparticles [15,16].

Animal models are often used to investigate pulmonary fibrosis, and they play an important role in understanding the pathogenesis of this disease. Bleomycin (BLM) is an anticancer agent with direct DNA strand breakage and interruption of the cell cycle. However, one of the major side effects of BLM therapy is pulmonary fibrosis, which is mediated by damage caused by a low level of hydrolase produced to inactivate BLM in lungs [17]. On this account, BLM is often used to generate experimental animal models of pulmonary fibrosis [18,19,20]. Using C57BL/6 mice implanted with an osmotic minipump, Harrison and colleagues demonstrated that continuous subcutaneous infusion of BLM over one week resulted in chronic, progressive and extensive pulmonary fibrosis [21]. Focal fibrotic lesions were mainly found in the sub-pleural area at Day 14 after BLM treatment, which expanded later to the central regions of the lung parenchyma at Day 21 [22,23].

On consideration that future studies on the mechanism could be supported by a series of available transgenic mice, mice were used in the present study to evaluate the effects of pulmonary exposure to ZnO nanoparticle. Mice treated with BLM (the BLM groups) and non-BLM-treated mice (the SALINE groups) were exposed to ZnO nanoparticles suspension or vehicle medium by pharyngeal aspiration. The tested hypothesis is that BLM-treated mice are susceptible to exogenous stimuli and exposure, so that exposure to ZnO nanoparticles accelerates or enhances pulmonary fibrosis induced by BLM treatment. In the first experiment, pulmonary effects were examined at 10 days after administration to see the possible acceleration of pulmonary fibrosis, which was progressive at Days 14 and 21 in a previous study [22]. However, a fibrotic lesion induced by BLM was not found at Day 10 after ZnO exposure, although severe inflammation was induced by ZnO nanoparticles, and ZnO exposure and BLM treatment were found to increase profibrotic cytokines synergistically. Therefore, the second experiment was conducted to evaluate how exposure to ZnO nanoparticles modifies the degree of fibrosis induced by BLM at Day 14.

## 2. Results

### 2.1. Characterization of ZnO Nanoparticles

The surface area of the primary ZnO nanoparticles was 50.72 m^2^/g, as measured by the Brunauer–Emmett–Teller (BET) gas absorption technique. No endotoxin was detected when the particles were suspended in distilled water. Dynamic light scattering (DLS) showed the aggregation of the nanoparticles in the dispersion medium (DM) with an average hydrodynamic size of 153.3 ± 1.0 nm. The presence of nano-sized particles was confirmed in the medium: the numbers of particles of less than 91.28 and 105.7 nm were 31.2% ± 0.8% and 71.2% ± 1.7%, respectively; the volume of particles of less than 91.28 and 105.7 nm were 19.6% ± 0.8% and 54.6% ± 2.1%, respectively.

### 2.2. Effects of BLM and ZnO Nanoparticles on Body and Lung Weights

Body weight decreased after pharyngeal aspiration in the SALINE group, but started to recover from Days 2 to 5 after administration. On the other hand, body weight diminished continuously in the BLM groups at both ZnO exposure levels. Moreover, the severity of body weight loss and the decreased activity level in mice exposed to ZnO nanoparticles were dose dependent. Four out of seven mice died after Day 5 following exposure to 30 µg of ZnO nanoparticles.

Table 1 shows the body weight and relative lung weight (lung weight (mg) divided by body weight (g)). In the SALINE groups, body weight was lower, while relative lung weight was higher in the 30 µg of ZnO-exposed mice compared with the vehicle control. A similar trend was observed in the BLM groups.

**Table 1:** Comparison of body weight and relative lung weight at Day 10 after administration. Data are the mean ± SD; * *p* < 0.05; ** *p* < 0.01; compared with the vehicle control. ^a^ The relative lung weight is the value of the lung weight (mg) divided by the body weight (g). BLM, bleomycin; ZnO, zinc oxide.

Subcutaneous Infusion	SALINE			BLM		
Groups	No ZnO	10 µg ZnO	30 µg ZnO	No ZnO	10 µg ZnO	30 µg ZnO
Number of mice	6	7	6	6	6	7
Body weight (g)	22.65 ± 0.53	22.36 ± 0.81	21.03 ± 1.36 *	19.17 ± 0.49	17.99 ± 0.38 *	15.63 ± 0.74 **
Relative lung weight ^a^	9.90 ± 1.26	11.52 ± 1.39	18.04 ± 1.73 **	14.33 ± 1.95	15.90 ± 0.91	18.91 ± 0.64 **

### 2.3. Effects of ZnO Nanoparticles on Lung Histopathology

**Figure 1:**
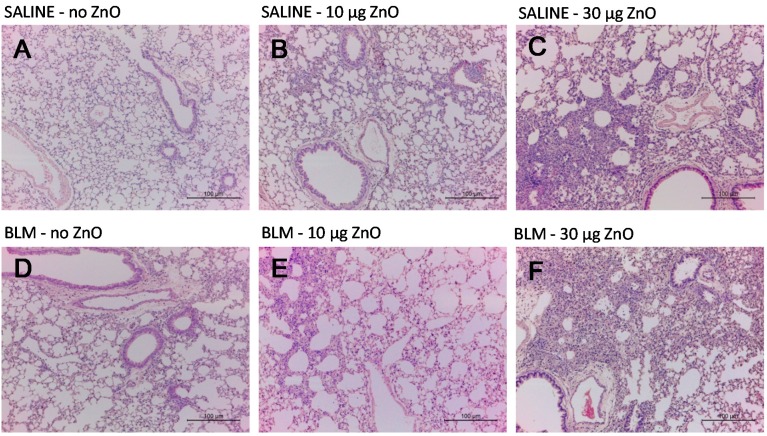
Representative optical micrographs of H&E-stained lung tissues. Mice were exposed to: (**A**) no ZnO; (**B**) 10 µg of ZnO; (**C**) 30 µg of ZnO nanoparticles without BLM treatment; (**D**) no ZnO; (**E**) 10 µg of ZnO; and (**F**) 30 µg of ZnO nanoparticles with BLM treatment. Samples were collected at Day 10 after administration.

**Figure 2:**
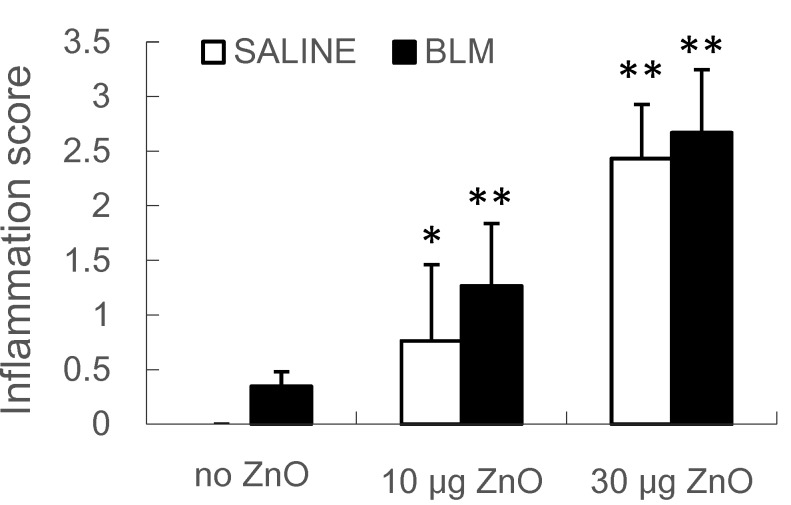
The results of histological scoring for total lung inflammation. Mice were exposed to ZnO nanoparticles with or without BLM treatment. Total lung inflammation at Day 10 after administration was defined using H&E staining of lung tissue. The number of mice was six in the SALINE-no ZnO, seven in the SALINE-10 µg of ZnO, six in the SALINE-30 µg of ZnO, six in the BLM-no ZnO, six in the BLM-10 µg of ZnO and seven in the BLM-30 µg of ZnO group. Data are the mean ± SD; * *p* < 0.05; ** *p* < 0.01; compared to the vehicle control.

Figure 1 shows the representative hematoxylin and eosin (H&E)-stained micrographs of the lungs sampled at 10 days after ZnO nanoparticle administration. Moderate to severe inflammatory infiltration in the peribronchiolar, peribronchial and perivascular area was observed in mice exposed to 10 µg or 30 µg of ZnO nanoparticles in the SALINE groups, respectively. Similar changes were found in the BLM groups with the corresponding ZnO exposure level. The dose-dependently increased pulmonary inflammation induced by ZnO nanoparticles was also demonstrated by the total inflammation score showed in Figure 2. However, no distinct collagen deposition was observed in the lungs with Masson’s trichrome staining (data not shown).

### 2.4. Effects of ZnO Nanoparticles on BALF Cytology and Cytokine Concentrations

**Figure 3:**
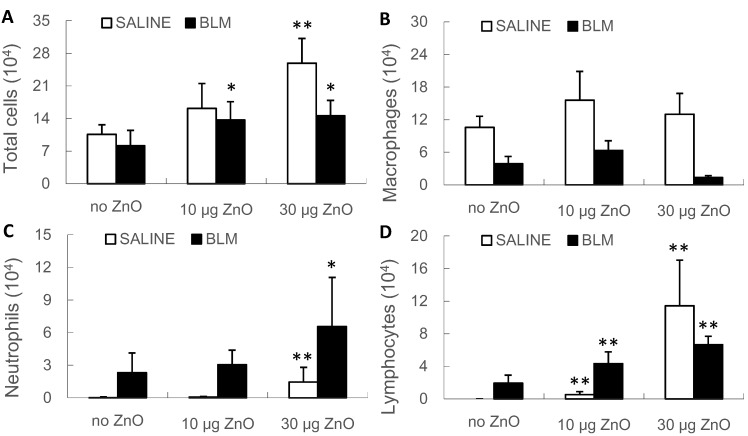
Total and differential cell counts in bronchoalveolar lavage fluid (BALF). Mice were exposed to ZnO nanoparticles with or without BLM treatment. The number of (**A**) total cells; (**B**) macrophages; (**C**) neutrophils and (**D**) lymphocytes in BALF were counted at Day 10 after administration. The number of mice was six in the SALINE-no ZnO, seven in the SALINE-10 µg of ZnO, six in the SALINE-30 µg of ZnO, six in of BLM-no ZnO, six in the BLM-10 µg of ZnO and seven in the BLM-30 µg of ZnO group. Data are the mean ± SD; * *p* < 0.05; ** *p* < 0.01; compared to the vehicle control.

BALF total cell, neutrophil and lymphocyte counts increased in a dose-dependent manner after administration of ZnO nanoparticles, in both the SALINE and BLM groups, compared with the vehicle control (Figure 3). The results of quantitative analysis were consistent with the pathological findings.

**Figure 4:**
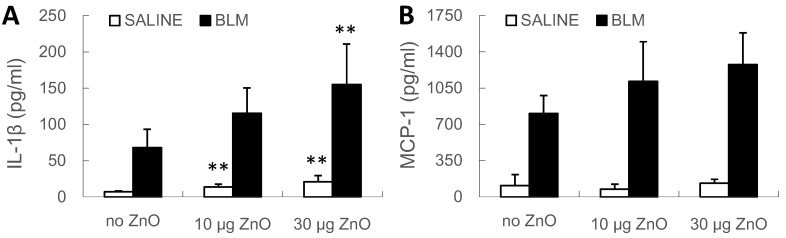
Cytokine levels in BALF. Mice were exposed to ZnO nanoparticles with or without BLM treatment. (**A**) IL-1β and (**B**) MCP-1 levels in BALF were determined at Day 10 after administration. The number of mice was six in the SALINE-no ZnO, seven in the SALINE-10 µg of ZnO, six in the SALINE-30 µg of ZnO, six in the BLM-no ZnO, six in the BLM-10 µg of ZnO and seven in the BLM-30 µg of ZnO group. Data are mean ± SD; ** *p* < 0.01; compared to vehicle control.

At Day 10, the level of interleukin (IL)-1β in BALF increased following exposure to 10 or 30 µg of ZnO nanoparticles, in the SALINE groups, and also following 30 µg of ZnO nanoparticles, in both the SALINE and BLM groups (Figure 4A). No significant change of the levels of monocyte chemotactic protein (MCP)-1 was detected, but an increasing trend induced by the exposure to ZnO nanoparticles was observed in BLM groups (Figure 4B). On the other hand, no ZnO-induced change of transforming growth factor (TGF)-β levels was found in the SALINE or BLM groups (data not shown).

### 2.5. Interaction between the BLM Treatment and ZnO Exposure

The interaction between the treatment with BLM and exposure to ZnO nanoparticles was tested by the analysis of covariance (ANCOVA). When such an interaction was significant, regression analysis for ZnO exposure level was applied separately using data of both the SALINE or BLM groups. At Day 10, the interaction between BLM treatment and ZnO exposure was significant for body weight and IL-1β and MCP-1 levels in BALF. Furthermore, the absolute values of the coefficients of body weight (two-fold) and the levels of IL-1β (seven-fold) and MCP-1 (14-fold) in BALF were higher in the BLM group than the SALINE group, suggesting the synergistic effects of BLM and ZnO nanoparticles on these parameters (Table 2).

**Table 2:** The coefficient of the regression line analysis for the SALINE and BLM groups at Day 10 after administration. SEM, standard error of the mean; IL-1β, interleukin-1β; MCP-1, monocyte chemotactic protein-1.

Parameters	SALINE		BLM	
	Coefficient ± SEM	*p*-Value	Coefficient ± SEM	*p*-Value
Body weight (g)				
ZnO exposure level (×10^−1^/µg)	−0.56 ± 0.18	0.007	−1.18 ± 0.11	<0.001
IL-1β (µg/mL)				
ZnO exposure level (per µg)	0.44 ± 0.11	0.725	2.90 ± 0.85	0.005
MCP-1 (µg/mL)				
ZnO exposure level (per µg)	1.11 ± 1.44	0.453	15.76 ± 7.02	0.043

### 2.6. Status at Day 14 after Administration

The dose of 30 µg for each mouse weighing 19–22 g in the 10-day experiment corresponded with the dose of 310 µg for rats weighing 200–250 g in a previous study [16]. No mortality was mentioned in the rat experiment, but exposure to ZnO at 30 µg resulted in high mortality in BLM-treated mice. Based on this finding, the high dose of ZnO nanoparticles was adjusted to 20 µg for the 14-day experiment.

**Figure 5:**
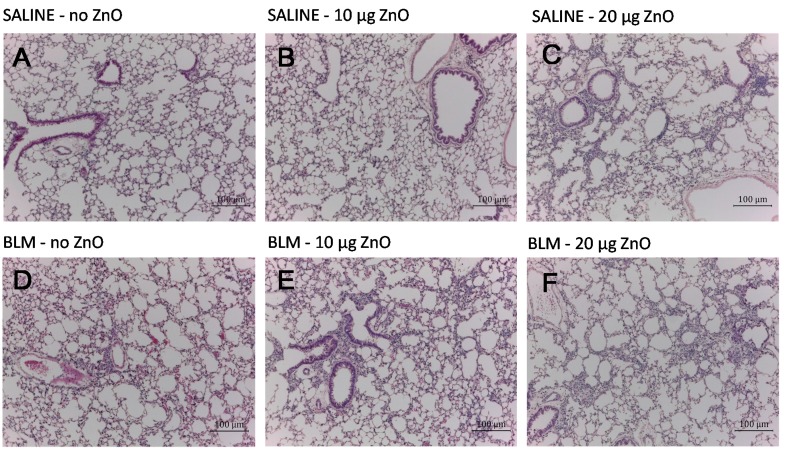
Representative optical micrographs of H&E-stained lung tissues. Mice were exposed to: (**A**) no ZnO; (**B**) 10 µg of ZnO; (**C**) 20 µg of ZnO nanoparticles without BLM treatment; (**D**) no ZnO; (**E**) 10 µg of ZnO and (**F**) 20 µg of ZnO nanoparticles with BLM treatment. Samples were collected at Day 14 after administration.

The recovery of pulmonary inflammation was observed at 14 days after administration. In the SALINE group, inflammatory infiltration in the alveolar septum recovered to normal lung architecture in mice exposed to 10 µg of ZnO nanoparticles, while a slight accumulation of inflammatory cells was noted in mice exposed to 20 µg of ZnO nanoparticles. In contrast, mild inflammation was evident in all mice of the BLM groups after exposure to ZnO nanoparticles (Figure 5 and Figure S1A). To check pulmonary fibrosis, Masson’s trichrome staining and alpha smooth muscle actin (α-SMA) immunohistochemistry were conducted using samples harvested on Day 14. It turns out that exposure to ZnO nanoparticles up to 20 µg had no obvious effects on collagen deposition or fibroblast proliferation, both in the SALINE and BLM groups (data not shown).

**Figure 6:**
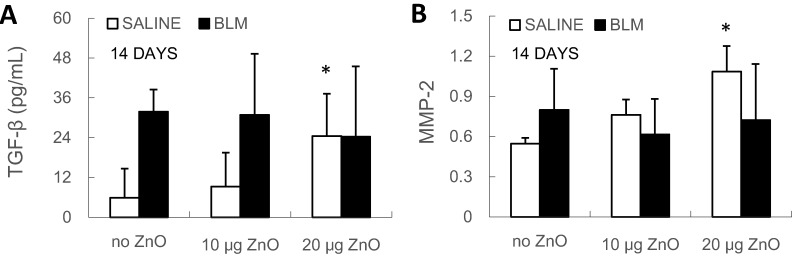
(**A**) TGF-β level in BALF and (**B**) MMP-2 relative mRNA expression level in lung tissue were determined at Day 14 after administration. Mice were exposed to ZnO nanoparticles with or without BLM treatment. The number of mice was six in the SALINE-no ZnO, seven in the SALINE-10 µg of ZnO, six in the SALINE-20 µg of ZnO, six in the BLM-no ZnO, six in the BLM-10 µg of ZnO and seven in the BLM-20 µg of ZnO group. Data are the means ± SD; * *p* < 0.05; compared to the vehicle control.

With regard to the biochemical changes, the level of transforming growth factor (TGF)-β in BALF and the relative mRNA expression level of matrix metalloproteinase (MMP)-2 in lung tissue were found to increase in the SALINE group after exposure to 20 µg of ZnO nanoparticles (Figure 6). However, there were no ZnO-induced changes in collagen I, collagen III, MMP-2, MMP-9, tissue inhibitor of MMPs (TIMP)-1, TIMP-2 and fibroblast specific protein (FSP)-1 mRNA levels in either the SALINE or BLM groups (Figure S2). Moreover, hydroxyproline content was analyzed in the lung samples of the BLM groups of the 14-day experiment, but no significant change was found after exposure to ZnO nanoparticles (Figure S1B).

## 3. Discussion

The present study demonstrated that pharyngeal aspiration of ZnO nanoparticles induced severe, but transient, pulmonary inflammation. No distinct fibrotic changes in the lungs were observed in the sub-acute phase after a bolus of ZnO nanoparticles in mice, but subcutaneous infusion of BLM and pulmonary exposure to ZnO nanoparticles synergistically increased the concentrations of pro-fibrotic cytokines in BALF from the lungs.

At 10 days after administration, marked inflammatory cell infiltration and thickening of alveolar septa was observed histopathologically, in association with high total cell, neutrophil and lymphocyte counts and upregulation of IL-1β in BALF after exposure to ZnO nanoparticles. Notably, IL-1β and MCP-1 levels were higher in BLM-treated mice than non-treated control mice at each ZnO exposure level, and a synergistic effect of BLM treatment and ZnO exposure was detected. IL-1β and MCP-1 are considered to promote pulmonary fibrosis by triggering the activation and proliferation of fibroblasts and to stimulate collagen production [24,25,26]. Although the pathological findings in BLM-treated and non-treated mice were similar and there was no fibrotic change at Day 10 after exposure, the effects of treatment with BLM and exposure to ZnO were synergistic. Based on this result, we conducted another set of animal experiments in which we examined the effects at 14 days after exposure to ZnO, in order to examine whether exposure to ZnO nanoparticles enhances the fibrotic effects of pulmonary fibrosis induced by treatment with BLM. The observation at Day 14 was considered to be appropriate for the examination, as fibrotic change was progressive at Days 14 and 21 in the previous study when mice received BLM with a lower dose of 62.5 mg/kg of body weight [22]. Unexpectedly, however, the pathological changes illustrated marked recovery of inflammatory infiltration at Day 14 in both BLM non-treated and BLM-treated mice. This transient inflammation induced by pharyngeal aspiration of ZnO nanoparticles is compatible with the well-known metal fume fever: the most frequently-described systemic illness in welders. Influenza-like symptoms typically appear within 4–12 h after inhalation of fumes and resolve within 24–48 h [27,28].

In mice without BLM treatment, exposure to 20 µg of ZnO nanoparticles increased the BALF TGF-β level and the mRNA expression levels of MMP-2 in the lung tissue at Day 14. TGF-β and MMP-2 play crucial roles in the development of pulmonary fibrosis [29], but exposure to ZnO nanoparticles showed no effects on mRNA expression levels of collagen I and III in the lung tissue. Such a minimal fibrotic effect of ZnO nanoparticles found in the present study is consistent with previous studies: Chang *et al.* described normal lung histology without fibrosis at Day 28 after intratracheal instillation of 80 µg of ZnO nanoparticles [30], and Adamcakova-Dodd *et al.* found only small changes in inflammatory parameters and rare pathological changes with exposure to ZnO nanoparticles of up to 13 weeks of inhalation [31]. However, a previous study on rats exposed to 310 µg of ZnO nanoparticles reported detection of high TGF-β levels in BALF at 24 h, 1 week and 4 weeks after intratracheal instillation and collagen deposition in lung sections at 4 weeks after administration [16]. The discrepancy in the fibrotic changes between mice and rats could represent species differences in the pulmonary responses to ZnO nanoparticles. A similar outcome was described in rodents exposed to ultrafine titanium dioxide particles: Rats developed severer inflammatory response compared to mice [32]. A case report showed that welders exposed to condensation aerosol with high ZnO concentrations developed pneumoconiosis with exogenous fibrosing alveolitis [33], indicating the risk for individuals to develop pulmonary fibrosis after long-term exposure to ZnO nanoparticles, but there are very few animal studies that have evaluated the profibrotic effect of pulmonary exposure to ZnO nanoparticles. On account of the species difference mentioned above, the fibrotic effect of pulmonary exposure to ZnO nanoparticles remains unclear.

In BLM-treated mice, all parameters related to inflammation and fibrosis were not different between ZnO-exposed and non-exposed mice at Day 14, suggesting the attenuation of the effects of pharyngeal aspiration of ZnO nanoparticles over time. Moreover, the fibrotic changes did not seem to be promoted by a single exposure to ZnO nanoparticles at this time point. As a limitation of this study, pharyngeal aspiration or intratracheal instillation, a method of delivery routinely used for the exposure of animals to particles, is a less physiologic method of exposure compared with inhalation and may alter the study results or sensitivity early after administration [34]. Additionally, the first experiment showed high mortality in BLM-treated group when mice were exposed to 30 μg of ZnO nanoparticles, which might indicate the problem of the present mouse model combining BLM treatment with bolus administration of ZnO nanoparticles. It is worth trying a continuous inhalation experiment, as it better mimics the exposure situation in humans and might result in persistent irritation or inflammation in animals, leading to a continuous interaction between BLM treatment and ZnO exposure.

The dissolved proportion of ZnO nanoparticles in the suspensions was not examined in our experiments, which is another limitation of the present study. Solubility is considered one of the most important properties affecting the biological effects in the safety evaluation of ZnO nanoparticles [2,8]. Nonetheless, the dissolved Zn^2+^ content did not seem to completely explain the toxicity of ZnO nanoparticles. In a previous study, another research group prepared a ZnCl_2_ solution, which contained the same concentration of Zn^2+^ as the ZnO nanoparticles suspension, and both were intratracheally instilled to rats [14]. As a result, the oxidative stress induced by the particle suspension was stronger than that of the ZnCl_2_ solution. This is supported by the result that the released Zn^2+^ in the cell culture medium was estimated to contribute 10% of the effects to the cells [35]. Besides, different inflammatory effects were identified in cultured cells and rat lungs exposed to ZnO nanoparticles or their aqueous extracts [36]. The results obtained in the present study should be considered based on the understanding that the toxicity of ZnO nanoparticles derived from both the nanoparticle itself and the ionized proportion. Further study of the mechanism of the ionized proportion of ZnO nanoparticles should be conducted in the future.

## 4. Experimental Section

### 4.1. ZnO Nanoparticles

ZnO nanoparticles (MKN-ZnO-020; mkNano, Mississauga, ON, Canada) with a primary diameter of 20 nm were used in the present study. BET was used to measure the surface area (Macsorb HM model-1201, MOUNTECH, Tokyo, Japan). Endotoxin analysis was conducted using the Pierce LAL Chromogenic Endotoxin Quantitation Kit (Thermo Scientific, Waltham, MA, USA). For the animal experiments, suspensions of ZnO nanoparticles were prepared in a biocompatible dispersion medium (DM) containing albumin, surfactant and phosphate-buffered saline [37]. A cup-type sonicator was used to disperse the nanoparticles, as described previously by our laboratory [38]. Briefly, ZnO nanoparticles were dispersed at 100 W, 80% pulse mode for 10 min. After sonication, size characterization was conducted using DLS (Zetasizer Nano-S; Malvern Instruments, Worcestershire, UK). The sonicated suspension of ZnO nanoparticles was used for animal treatment at 1 to 3 days after sonication. The stability of the suspension after sonication was confirmed: hydrodynamic sizes only increased 1.9% and 3.4% at 1 and 7 days after sonication, when compared with the sizes measured at the same day of sonication.

### 4.2. Animals

Nine-week-old female C57BL/6J mice weighing 19–22 g were purchased from CLEA Japan Inc. (Tokyo, Japan). All mice were housed and acclimatized to the new environment for 1 week in a pathogen-free animal room at temperature (23–25 °C)- and humidity (55%–60%)-controlled conditions. Light was set with a 12-h light-dark cycle (on at 09:00 and off at 21:00), and food and water were provided *ad libitum*. All experimental procedures were performed in accordance with institutional guidelines for animal research, and the study was approved by the animal ethics committee of Mie University.

Mice were anesthetized with pentobarbital and then exposed to 40-µL aliquot of samples of ZnO nanoparticles by pharyngeal aspiration [39]. The technique of pharyngeal aspiration involved placement of the nanoparticle suspension on the back of the tongue followed by pulling of the tongue to induce a reflex gasp with resultant aspiration of the droplets. In both BLM and SALINE groups, mice received either DM (vehicle control), 10 or 30 µg of ZnO nanoparticles in the 10-day experiment; while in the 14-day experiment, mice received either DM, 10 or 20 µg of ZnO nanoparticles.

According to the American Conference of Governmental Industrial Hygienists, the threshold limit value (TLV) for ZnO particles is 2 mg/m^3^ (time-weighted average). The amount of ZnO inhaled in one week by an adult will then be calculated as 0.48 mg/kg of body weight (500 mL air/breath, 12 breath/min, 8 h/day, 40 h/week) [40]. In the present study, mice were exposed to 0.5, 1.0 or 1.5 mg/kg of body weight ZnO nanoparticles, so that the lowest dose was comparable to the deposition from the inhalation to ZnO at the TLV level for one week.

After pharyngeal aspiration, mice were treated with BLM. Mice were divided at random into the BLM and SALINE groups. In the BLM groups, lung injury was induced by 7-day constant subcutaneous infusion of bleomycin sulfate (Nihon Kayaku, Tokyo, Japan) at 100 mg/kg of body weight, dissolved in sterile saline using osmotic minipumps (Model 2001; Alzet Corporation, Palo Alto, CA, USA). Minipumps only loaded with saline were placed in the SALINE groups [41]. The number of mice was 6 in the SALINE-no ZnO, 7 in the SALINE-10 µg of ZnO, 6 in the SALINE-30 µg of ZnO, 6 in the BLM-no ZnO, 6 in the BLM-10 µg of ZnO and 7 in the BLM-30 µg of ZnO group in the 10-day experiment, while 6 in the SALINE-no ZnO, 6 in the SALINE-10 µg of ZnO, 7 in the SALINE-30 µg of ZnO, 6 in the BLM-no ZnO, 6 in the BLM-10 µg of ZnO and 6 in the BLM-30 µg of ZnO group in the 14-day experiment.

At 10 and 14 days after administration, mice were sacrificed by i.p. injection of pentobarbital. Bronchoalveolar lavage (BAL) was performed by cannulating the trachea with an 18-gauge needle, and infusion of 2 mL of saline three times followed. The perfused lungs were then dissected out, and the left lung was fixed in 4% phosphate-buffered paraformaldehyde (PFA), while the right lungs were frozen on dry ice and subsequently stored at −80 °C for later analysis.

### 4.3. Histopathological Examination

The left lung was fixed in 4% PFA for 24–30 h and then treated with 70% ethanol for several days within 1 week. After routine dehydration and paraffin embedding, 3 µm-thick sections were cut and stained with: (1) H&E staining to evaluate inflammation and general pathological change; (2) Masson’s trichrome staining to detect collagen deposition; and (3) α-SMA immunohistochemistry to assess fibroblast proliferation, which was performed with an ImmPACT™ BAD Peroxidase Substrate Kit (Vector, Burlingame, CA, USA) using anti-α-SMA rabbit polyclonal antibodies (Abcam, Cambridge, UK). Tissue slices were examined under an optical microscope (Model DM750, Leica Microsystems, Wetzlar, Germany), and images were captured with the Leica Application Suite V3 software. Lung inflammation was scored in a blinded fashion using a reproducible scoring system described elsewhere [42]. The degree of peribronchial and perivascular inflammation was evaluated on a subjective scale of 0 to 3 (0: no detectable inflammation; 1: occasional cuffing with inflammatory cells; 2: most bronchi or vessels surrounded by a thin layer of inflammatory cells; 3: most bronchi or vessels surrounded by a thick layer of inflammatory cells). Total lung inflammation was defined as the average of the peribronchial and perivascular inflammation scores based on 3 lung sections per mouse.

### 4.4. Total and Differential Cell Count in BALF

The recovered BALF was centrifuged (1000× *g*, 10 min, 4 °C), and the supernatant was stored immediately at −80 °C until analysis. The cell pellet was resuspended for total and differential cell count. The total cell count was measured using ChemoMetec nucleocounter (ChemoMetec A/S, Allerød, Denmark) [43], and the cell smear was stained with May-Grunwald-Giemsa (Merck, Darmstadt, Germany) for differential cell count: the number of cells was counted in 10 fields (20× magnification) of each slide.

### 4.5. BALF Biochemical Analysis

The levels of IL-1β, MCP-1 and TGF-β in BALF were analyzed by enzyme-linked immunosorbent assay (ELISA) using commercially available ELISA kits (eBioscience, Inc., San Diego, CA, USA). Assays were performed in duplicate wells, and the absorbance at 450 nm was measured with a microtiter plate reader.

### 4.6. Real-Time Quantitative Reverse Transcription-Polymerase Chain Reaction (RT-PCR)

The mRNA expression levels of collagen I, collagen III, MMP-2, 9, TIMP-1, 2 and FSP-1 in lung tissues were determined by RT-PCR. About 15 mg of frozen lung tissues were homogenized, and total RNA was isolated using the ReliaPrep™ RNA Tissue Miniprep System (Promega, WI, USA) and stored at −80 °C until reverse transcription. The purity and concentration of the obtained RNA were determined with a Nanodrop-1000 3.5.1 (Nanodrop, Inc., Wilmington, DE, USA). The SuperScript III Reverse transcriptase kit (Life Technologies, Carlsbad, CA, USA) was used to convert the RNA to complementary DNA (cDNA), which was stored at −30 °C until quantification by real-time PCR (M×3005P QPCR System, Agilent Technologies, Waldbronn, Germany). The relative levels of mRNA expression were normalized to β-actin for each gene. The primers and probes were designed by the Universal ProbeLibrary Assay Design Center (Roche Diagnostics, Basel, Switzerland).

### 4.7. Hydroxyproline Content

To assess the amount of collagen, about 10 mg of homogenized tissue sample was hydrolyzed in 100 µL 10 N hydrochloric acid at 120 °C for 24 h. Hydroxyproline content was measured using a hydroxyproline assay kit (Chondrex, Inc., Redmond, WA, USA) according to the instructions provided by the manufacturer.

### 4.8. Statistical Analysis

Data were expressed as the means ± standard deviation. Multiple comparisons between the ZnO exposure groups and the vehicle control groups were performed using Dunnett’s multiple comparison method following one-way ANOVA. Kruskal–Wallis tests were used when the variances were still regarded as heterogeneous by Levene’s test after a logarithm or square root transformation. ANCOVA was performed to examine the effects of BLM treatment (factor) and the trend with ZnO exposure levels (covariate). When the interaction was significant, regression analysis on ZnO exposure level was applied in either SALINE or BLM groups, separately. Statistical analyses were performed with SPSS 20 software (IBM Corporation, New York, NY, USA). A *p*-value less than 0.05 was considered as statistically significant.

## 5. Conclusions

Pharyngeal aspiration of ZnO nanoparticles in mice resulted in transient infiltration of inflammatory cells and upregulation of inflammatory cytokines in the lungs. The synergistic effect of pulmonary exposure to ZnO nanoparticles and subcutaneous infusion of BLM on the secretion of pro-fibrotic cytokines in the lungs was demonstrated, although a single bolus of ZnO nanoparticles did not induce distinct pulmonary fibrotic changes in mice.

## Supplementary Materials

Supplementary figures can be found at: http://www.mdpi.com/1422-0067/16/01/0660/s1.

## References

[1] Donaldson K., Stone V., Tran C.L., Kreyling W., Borm P.J. (2004). Nanotoxicology. Occup. Environ. Med..

[2] Nel A., Xia T., Mädler L., Li N. (2006). Toxic potential of materials at the nanolevel. Science.

[3] Becheri A., Dürr M., Nostro P.L., Baglioni P. (2008). Synthesis and characterization of zinc oxide nanoparticles: Application to textiles as UV-absorbers. J. Nanopart. Res..

[4] Rekha K., Nirmala M., Nair M.G., Anukaliani A. (2010). Structural, optical, photocatalytic and antibacterial activity of zinc oxide and manganese doped zinc oxide nanoparticles. Phys. B.

[5] Raghupathi K.R., Koodali R.T., Manna A.C. (2011). Size-dependent bacterial growth inhibition and mechanism of antibacterial activity of zinc oxide nanoparticles. Langmuir.

[6] Oberdörster G., Oberdörster E., Oberdörster J. (2005). Nanotoxicology: An emerging discipline evolving from studies of ultrafine particles. Environ. Health Perspect..

[7] Kim Y.H., Fazlollahi F., Kennedy I.M., Yacobi N.R., Hamm-Alvarez S.F., Borok Z., Kim K.J., Crandall E.D. (2010). Alveolar epithelial cell injury due to zinc oxide nanoparticle exposure. Am. J. Respir. Crit. Care Med..

[8] Krug H.F., Wick P. (2011). Nanotoxicology: An interdisciplinary challenge. Angew. Chem. Int. Ed. Engl..

[9] Akhtar M.J., Ahamed M., Kumar S., Khan M.M., Ahmad J., Alrokayan S.A. (2012). Zinc oxide nanoparticles selectively induce apoptosis in human cancer cells through reactive oxygen species. Int. J. Nanomed..

[10] Kang T., Guan R., Chen X., Song Y., Jiang H., Zhao J. (2013). In vitro toxicity of different-sized ZnO nanoparticles in Caco-2 cells. Nanoscale Res. Lett..

[11] Sahu D., Kannan G.M., Vijayaraghavan R., Anand T., Khanum F. (2013). Nanosized zinc oxide induces toxicity in human lung cells. ISRN Toxicol..

[12] Wilhelmi V., Fischer U., Weighardt H., Schulze-Osthoff K., Nickel C., Stahlmecke B., Kuhlbusch T.A., Scherbart A.M., Esser C., Schins R.P. (2013). Zinc oxide nanoparticles induce necrosis and apoptosis in macrophages in a p47phox- and Nrf2-independent manner. PLoS One.

[13] Warheit D.B., Sayes C.M., Reed K.L. (2009). Nanoscale and fine zinc oxide particles: Can in vitro assays accurately forecast lung hazards following inhalation exposures?. Environ. Sci. Technol..

[14] Fukui H., Horie M., Endoh S., Kato H., Fujita K., Nishio K., Komaba L.K., Maru J., Miyauhi A., Nakamura A. (2012). Association of zinc ion release and oxidative stress induced by intratracheal instillation of ZnO nanoparticles to rat lung. Chem. Biol. Interact..

[15] Cho W.S., Duffin R., Poland C.A., Howie S.E., MacNee W., Bradley M., Megson I.L., Donaldson K. (2010). Metal oxide nanoparticles induce unique inflammatory footprints in the lung: Important implications for nanoparticle testing. Environ. Health Perspect..

[16] Cho W.S., Duffin R., Howie S.E., Scotton C.J., Wallace W.A., Macnee W., Bradley M., Megson I.L., Donaldson K. (2011). Progressive severe lung injury by zinc oxide nanoparticles; the role of Zn^2+^ dissolution inside lysosomes. Part. Fibre Toxicol..

[17] Moeller A., Ask K., Warburton D., Gauldie J., Kolb M. (2008). The bleomycin animal model: A useful tool to investigate treatment options for idiopathic pulmonary fibrosis?. Int. J. Biochem. Cell Biol..

[18] Manali E.D., Moschos C., Triantafillidou C., Kotanidou A., Psallidas I., Karabela. S.P., Roussos C., Papiris S., Armaganidis A., Stathopoulos GT. (2011). Static and dynamic mechanics of the murine lung after intratracheal bleomycin. BMC Pulm. Med..

[19] Tsai K.D., Yang S.M., Lee J.C., Wong H.Y., Shih C.M., Lin T.H., Tseng M.J., Chen W. (2011). Panax notoginseng attenuates bleomycin-induced pulmonary fibrosis in mice. Evid. Based Complement. Alternat. Med..

[20] Bogatkevich G.S., Ludwicka-Bradley A., Nietert P.J., Akter T., van Ryn J., Silver R.M. (2011). Antiinflammatory and antifibrotic effects of the oral direct thrombin inhibitor dabigatran etexilate in a murine model of interstitial lung disease. Arthritis Rheumatol..

[21] Harrison J.H., Lazo J.S. (1987). High dose continuous infusion of bleomycin in mice: A new model for drug-induced pulmonary fibrosis. J. Pharmacol. Exp. Ther..

[22] Yasui H., Gabazza E.C., Tamaki S., Kobayashi T., Hataji O., Yuda H., Shimizu S., Suzuki K., Adachi Y., Taguchi O. (2001). Intratracheal administration of activated protein C inhibits bleomycin-induced lung fibrosis in the mouse. Am. J. Respir. Crit. Care Med..

[23] Aono Y., Nishioka Y., Inayama M., Ugai M., Kishi J., Uehara H., Izumi K., Sone S. (2005). Imatinib as a novel antifibrotic agent in bleomycin-induced pulmonary fibrosis in mice. Am. J. Respir. Crit. Care Med..

[24] Gharaee-Kermani M., McCullumsmith R.E., Charo I.F., Kunkel S.L., Phan S.H. (2003). CC-chemokine receptor 2 required for bleomycin-induced pulmonary fibrosis. Cytokine.

[25] Olman M.A., White K.E., Ware L.B., Simmons W.L., Benveniste E.N., Zhu S., Pugin J., Matthay M.A. (2004). Pulmonary edema fluid from patients with early lung injury stimulates fibroblast proliferation through IL-1 beta-induced IL-6 expression. J. Immunol..

[26] Dos Santos G., Kutuzov M.A., Ridge K.M. (2012). The inflammasome in lung diseases. Am. J. Physiol. Lung Cell Mol. Physiol..

[27] El-Zein M., Malo J.L., Infante-Rivard C., Gautrin D. (2003). Prevalence and association of welding related systemic and respiratory symptoms in welders. Occup. Environ. Med..

[28] Kelleher P., Pacheco K., Newman L.S. (2000). Inorganic dust pneumonias: The metal-related parenchymal disorders. Environ. Health Perspect..

[29] Lagente V., Manoury B., Nénan S., Le Quément C., Martin-Chouly C., Boichot E. (2005). Role of matrix metalloproteinases in the development of airway inflammation and remodeling. Braz. J. Med. Biol. Res..

[30] Chang H., Ho C.C., Yang C.S., Chang W.H., Tsai M.H., Tsai H.T., Lin P. (2013). Involvement of MyD88 in zinc oxide nanoparticle-induced lung inflammation. Exp. Toxicol. Pathol..

[31] Adamcakova-Dodd A., Stebounova L.V., Kim J.S., Vorrink S.U., Ault A.P., O’Shaughnessy P.T., Grassian V.H., Thorne P.S. (2014). Toxicity assessment of zinc oxide nanoparticles using sub-acute and sub-chronic murine inhalation models. Part. Fibre Toxicol..

[32] Bermudez E., Mangum J.B., Wong B.A., Asgharian B., Hext P.M., Warheit D.B., Everitt J.I. (2004). Pulmonary responses of mice, rats, and hamsters to subchronic inhalation of ultrafine titanium dioxide particles. Toxicol. Sci..

[33] Voznesenskiĭ N.K. (2004). Exogenous fibrosing alveolitis due to the condensation aerosol (smoke) of zinc oxide (abstract). Vestn. Ross. Akad. Med. Nauk.

[34] Osier M., Baggs R.B., Oberdörster G. (1997). Intratracheal instillation *versus* intratracheal inhalation: Influence of cytokines on inflammatory response. Environ. Health Perspect..

[35] Xu M., Li J., Hanagata N., Su H., Chen H., Fujita D. (2013). Challenge to assess the toxic contribution of metal cation released from nanomaterials for nanotoxicology—The case of ZnO nanoparticles. Nanoscale.

[36] Cho W.S., Duffin R., Poland C.A., Duschl A., Oostingh G.J., Macnee W., Bradley M., Megson I.L., Donaldson K. (2012). Differential pro-inflammatory effects of metal oxide nanoparticles and their soluble ions *in vitro* and *in vivo*; zinc and copper nanoparticles, but not their ions, recruit eosinophils to the lungs. Nanotoxicology.

[37] Porter D., Sriram K., Wolfarth M., Jefferson A., Schwegler-Berry D., Andrew M., Castranova V. (2008). A biocompatible medium for nanoparticle dispersion. Nanotoxicology.

[38] Wu W., Ichihara G., Suzuki Y., Izuoka K., Oikawa-Tada S., Chang J., Sakai K., Miyazawa K., Porter D., Castranova V. (2014). Dispersion method for safety research on manufactured nanomaterials. Ind. Health.

[39] Gabazza E.C., Kasper M., Ohta K., Keane M., D’Alessandro-Gabazza C., Fujimoto H., Nishii Y., Nakahara H., Takagi T., Menon A.G. (2004). Decreased expression of aquaporin-5 in bleomycin-induced lung fibrosis in the mouse. Pathol. Int..

[40] Scanlan C.L., Wilkins R., Stoller J.K. (1998). Egan’s Fundamentals of Respiratory Care.

[41] Porter D.W., Hubbs A.F., Mercer R.R., Wu N., Wolfarth M.G., Sriram K., Leonard S., Battelli L., Schwegler-Berry D., Friend S. (2010). Mouse pulmonary dose- and time course-responses induced by exposure to multi-walled carbon nanotubes. Toxicology.

[42] Braber S., Henricks P.A., Nijkamp F.P., Kraneveld A.D., Folkerts G. (2010). Inflammatory changes in the airways of mice caused by cigarette smoke exposure are only partially reversed after smoking cessation. Respir. Res..

[43] Boveda-Ruiz D., D’Alessandro-Gabazza C.N., Toda M., Takagi T., Naito M., Matsushima Y., Matsumoto T., Kobayashi T., Gil-Bernabe P., Chelakkot-Govindalayathil A.L. (2013). Differential role of regulatory T cells in early and late stages of pulmonary fibrosis. Immunobiology.

